# Microfluidic Paper-Based Sample Concentration Using Ion Concentration Polarization with Smartphone Detection

**DOI:** 10.3390/mi7110199

**Published:** 2016-11-04

**Authors:** Xue Li, Yanan Niu, Yunyi Chen, Di Wu, Long Yi, Xianbo Qiu

**Affiliations:** Institute of Microfluidic Chip Development in Biomedical Engineering, State Key Laboratory of Organic-Inorganic Composites, College of Information Science and Technology, Beijing University of Chemical Technology, Beijing 100029, China; 2015210307@grad.buct.edu.cn (X.L.); 2015210306@grad.buct.edu.cn (Y.N.); 2015200729@mail.buct.edu.cn (Y.C.); wudi@mail.buct.edu.cn (D.W.); yilong@mail.buct.edu.cn (L.Y.)

**Keywords:** ion concentration polarization (ICP), sample concentration, paper-based microfluidics, Nafion coating, smartphone, point-of-care test

## Abstract

A simple method for microfluidic paper-based sample concentration using ion concentration polarization (ICP) with smartphone detection is developed. The concise and low-cost microfluidic paper-based ICP analytical device, which consists of a black backing layer, a nitrocellulose membrane, and two absorbent pads, is fabricated with the simple lamination method which is widely used for lateral flow strips. Sample concentration on the nitrocellulose membrane is monitored in real time by a smartphone whose camera is used to collect the fluorescence images from the ICP device. A custom image processing algorithm running on the smartphone is used to track the concentrated sample and obtain its fluorescence signal intensity for quantitative analysis. Two different methods for Nafion coating are evaluated and their performances are compared. The characteristics of the ICP analytical device especially with intentionally adjusted physical properties are fully evaluated to optimize its performance as well as to extend its potential applications. Experimental results show that significant concentration enhancement with fluorescence dye sample is obtained with the developed ICP device when a fast depletion of fluorescent dye is observed. The platform based on the simply laminated ICP device with smartphone detection is desired for point-of-care testing in settings with poor resources.

## 1. Introduction

Ion concentration polarization (ICP) is an electrokinetic phenomenon caused by the transport of ions through ion-selective nanostructures [1]. As a technology for fast sample concentration with high flexibility, ICP has been used for water desalination, and separation and enrichment of charged bio-species with functionalized microchannels [2,3,4]. Because of ICP’s effect, ion depletion occurs in the region of ion-selective nanostructures, which accordingly cause ion enrichment at the boundary of ion depletion. ICP can be adopted into an integrated analytical device for the detection of biomolecules by separating, and concentrating DNA or protein from the lysed cells [5].

As a simple, rapid, easy-to-use and low-cost diagnosis method, the lateral flow immunoassay plays an important role in screening or rapid testing [6,7]. The lateral flow immunoassay can be integrated into microfluidic systems to detect the presence of antigens and antibodies to various pathogens [8,9]. Once the sample is applied onto the sample pad of the lateral flow strip, it will be specifically captured by the immobilized protein on the test line area when it migrates along the strip because of the capillary force. With the lateral flow on a nitrocellulose membrane, because of the capillary force, much simpler detection can be obtained compared to most other analytical methods [10]. Similar to the lateral flow strip test, microfluidic paper-based analytical devices (µPADs) rely on the capillary force to drive sample or reagent without active pumping [11,12]. With the characteristics of simplicity, versatility, low cost, ease of use, reagent storage, high throughput and disposability, µPADs have been regarded as ideal tools for point-of-care diagnostics in low-resource settings [13,14]. However, because of the natural limitation of the assays implemented on µPADs, their detection sensitivity still needs to be increased to improve both accuracy and specificity.

Recently, much effort has been made to increase the detection sensitivity of µPADs by adopting the ICP effect into paper-based microfluidic devices for sample concentration [15,16]. ICP normally occurs at the interface of microfluidic and ion-selective nanofluidic channels under an applied electric field [1], where a formed ion depletion region is able to repel the charged particles in the test sample continually toward a specific direction [17]. Various paper-based ICP devices are fabricated with different methods, for example wax printing, where wax is printed on paper with a wax printer and then heated to form paper-based channels where the applied sample is concentrated [18], and cutting, where each layer of the device including the channels is shaped in the desired dimensions and sizes with an electronic craft cutter or a computer-controlled blade [19]. In some cases, complicated lamination methods have to be adopted to fabricate the paper-based ICP devices [20], or paper-based ICP has to be performed with a trivial experimental setup [21], which is undesirable for point-of-care diagnosis whose essential features include simple and rapid detection, low cost and ease of operation.

Based on advanced embedded systems, smartphones provide a desired platform to implement point-of-care diagnosis with easy operation, cost-efficiency, convenience and portability [22,23]. With an integrated camera, various assays can be monitored in real time or at the end point by a smartphone when optical filters are incorporated if necessary [24,25]. The unique feature of auto-focus with a smartphone camera can significantly simplify the optical and mechanical design of the detection system. The standard function for camera control in the Java environment is beneficial to reduce the complexity of software development. Furthermore, the detection result can be conveniently transmitted by smartphone to other places, e.g., medical agencies for further analysis. As one of the potential solutions for point-of-care diagnostics, various smartphone-based microfluidic analytical devices are intensively being studied [26,27].

In this paper, we report a concise, simply fabricated, and easy-to-use microfluidic paper-based analytical device with an ICP effect for sample concentration. To obtain the ICP effect, two different methods are used to develop a Nafion-coated region on the sample channel, and their performances are evaluated and compared. To facilitate point-of-care diagnosis, the ICP process is monitored in real time by a smartphone camera and the collected fluorescent images are analyzed by a custom algorithm running on the smartphone for quantitative analysis. Paper-based ICP devices with intentionally adjusted physical properties are fabricated and evaluated to optimize their performance as well as to extend their application area. Experimental results with fluorescein isothiocyanate (FITC) show that significant concentration enhancement with the fluorescent dye sample on the developed microfluidic paper-based device is successfully achieved because of the ICP effect.

## 2. Materials and Methods

### 2.1. Design and Fabrication of Paper-Based ICP Device

**Figure 1:**
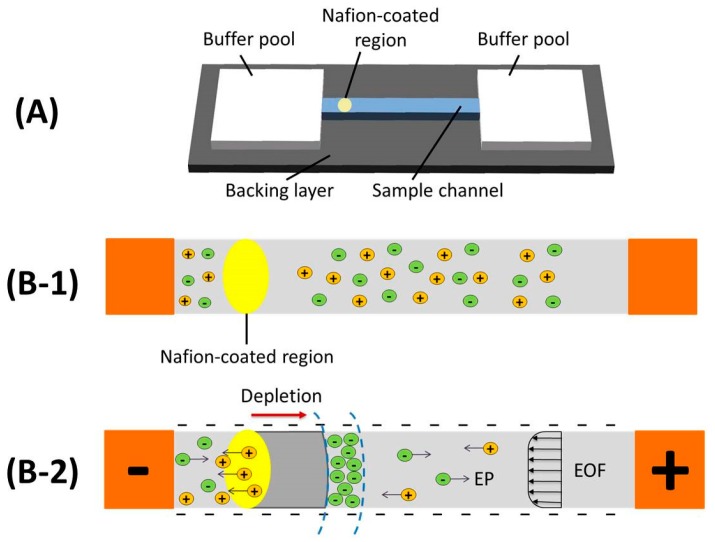
Design and operation of the paper-based ICP device. (**A**) Schematic of device with one sample channel, two buffer pools and one backing layer; (**B-1**) Schematic of non ICP state without an electrical field; (**B-2**) Schematic of ICP enrichment and depletion effects under applied voltage.

The developed paper-based ICP device consists of a sample channel made from nitrocellulose membrane with a thickness of 0.14 mm where a region coated with cation-selective nanoporous Nafion is developed at one end, and two buffer pools made from paper-based absorbent pads, and three of them are laminated with one another and then fixed on a black backing layer (polyvinyl chloride (PVC)) with one sided tape (Figure 1A). In the reported literature [16], it has been demonstrated that nitrocellulose membrane is a desired substrate for electrokinetic operations, for example ICP. Because nitrocellulose membrane is widely used in lateral flow immunoassay strips and µPADs, it will be much convenient for the ICP device made from nitrocellulose membrane to combine with those existed paper-based analytical devices to further improve their performance.

**Figure 2:**
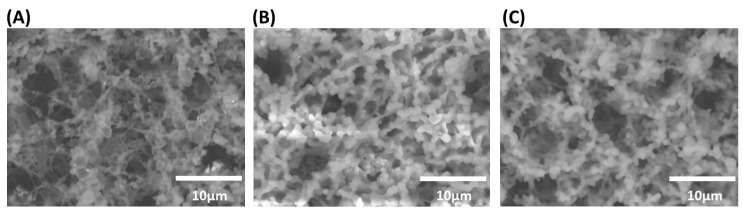
Scanning electron microscopy images of nitrocellulose membrane. (**A**) Without Nafion coating; (**B**) With Nafion coating (type I, without heating); (**C**) With Nafion coating (type II, with heating).

To operate the device, first enough DI water is added onto the two absorbent pads at both ends to establish two buffer pools. Once both absorbent pads are saturated with water, the middle nitrocellulose membrane will be prewetted by the DI water wicked from buffer pools because of the capillary force. And then, sample is added to the middle channel. As shown in Figure 1B-1, before a DC voltage is applied, there has no any ion enrichment. Nafion is an ion-selective material with negatively charged sulfonic groups [19]. Nafion containing nanopores on the nitrocellulose membrane substrate become ion-selective under the effects of an electrical field. In principle, the sulfonic acid groups in Nafion provide a net negative surface charge in the pores, which enables ion depletion by selectively transporting of cations through the Nafion-coated membrane during ICP [28,29]. Once ICP is induced because of DC electrical filed, cations are selectively gathered to form an ion enrichment zone around the boundary between the cathode and the Nafion-coated region (Figure 1B-2). Meanwhile, an ion depletion zone with electrical neutrality is being formed within the Nafion-coated region when anions are vacated. With the maintained DC electrical filed, the depletion zone propagates and drives anions toward the anode. Two major effects, the electrophoretic migration (EP) toward the anode and the electroosmotic flow (EOF) toward the cathode, both of which are determined by the distribution of both the applied electrical filed and the ionic concentration along the channel, dominate the net movement of anions in the channel. With lower ionic concentrations, higher EOF transport rates will be introduced and vice versa [30]. At one end of the channel close to the depletion zone, EP is higher than EOF, which drives anions to escape from the depletion zone and migrate in the anodic direction along the channel. At the other end of the channel close to the downstream of the depletion zone, EOF always drives the analytes to approach the depletion zone in the cathodic direction. Finally, the sample with negatively charged ions is focused at the depletion boundary by the two balanced opposing driving forces due to ICP effect.

Nanoporous Nafion is the key functional part in the paper-based analytical device to successfully induce ICP effect. To obtain desired nanoporous Nafion, it is important to find out a proper fabrication method to develop a Nafion-coated region on the ICP device. Therefore, as shown in Figure 2, two different methods for Nafion coating, which respectively corresponds to two types of paper-based ICP devices (type I and II), are evaluated and compared. The Nafion-coated nitrocellulose membrane was then fixed on a black backing layer with one side tape and then covered by two paper-based absorbent pads at two ends. The laminated ICP devices were covered in Petri dishes at room temperature before use.

The nitrocellulose membrane (HF-180, GE Whatman) with mean pore diameters of 6–8 µm was cut by a manual craft cutter to form a straight channel. Nafion perfluorinated resin solution (20 wt % in lower aliphatic alcohols and water, Sigma-Aldrich) was pipetted into each device with a size of 0.5 µL. After that, two different methods were respectively adopted to treat the nitrocellulose membrane. For ICP device of type I (Figure 2B), the nitrocellulose membrane was directly immersed in deionized (DI) water for 30 min to hydrate the Nafion, and then it was allowed to dry at room temperature. For ICP device of type II (Figure 2C), the nitrocellulose membrane was heated in an oven at a temperature of 150 °C for 30 s in order to cure the Nafion structure. The nitrocellulose membrane was then immersed in 10^−2^ M tris buffer for 1 min in order to rinse the Nafion for a higher efficiency of concentration, and finally it was left to dry at room temperature. As shown in Figure 2, the porous structure of the pristine nitrocellulose membrane is different from that of ICP device of type I or II. The patterns of Nafion-coated region respectively with ICP devices of type I and II are different from each other.

### 2.2. Experimental Setup with Smartphone Detection

**Figure 3:**
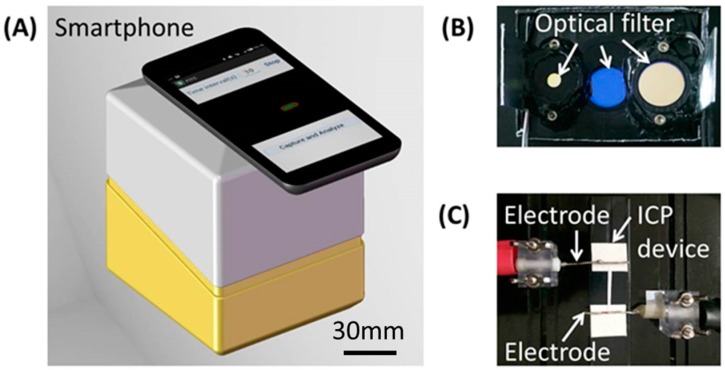
Experimental setup with smartphone detection. (**A**) Instrument box with a smartphone; (**B**) Optical module; (**C**) ICP device with applied electrodes.

As shown in Figure 3, a portable device was developed to perform experiments on a paper-based ICP device with smartphone detection. Two platinum electrodes with a diameter of 1 mm were used to apply a DC voltage to the paper-based ICP device from two buffer pools of the channel. Two LEDs with a specific wavelength were used to shine the sample through their own optical filters from the top, and the fluorescent signal of the concentrated sample was detected by collecting fluorescence images from the ICP device with a preset interval by a smartphone camera through another optical filter. All the components were assembled in an enclosed instrument box (100 mm × 90 mm × 100 mm) for desired optical detection except a smartphone fixed from the outside to monitor the ICP process. Once a DC voltage is applied to the ICP device, the process of sample concentration with ICP effect can be monitored in real-time through the control to the camera with custom application software running on the smartphone. The working current of LEDs can be adjusted with the application software to prevent signal saturation. At the current stage, a standard power supply was used to provide a DC voltage from outside of the instrument. In the future, alternative strategies, e.g., a small circuit module for voltage amplification, or a couple of batteries in series, can be adopted to replace the standard DC power supply in order to build a fully portable, low-cost and easy-to-use microfluidic ICP system for point-of-care diagnosis.

### 2.3. Image Processing for Quantitative Analysis

**Figure 4:**
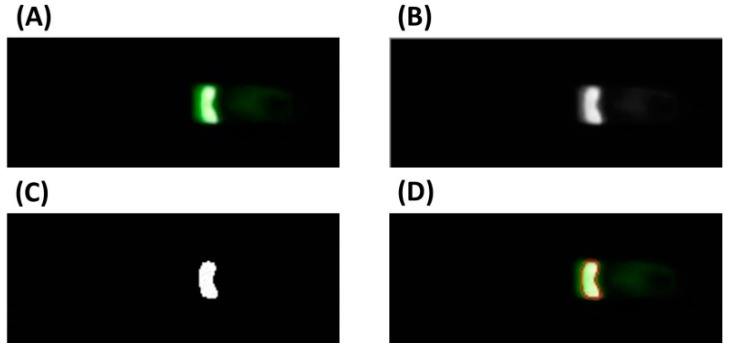
Image processing. (**A**) Original fluorescence image; (**B**) Converted gray image; (**C**) Converted binary image; (**D**) Marked fluorescence image.

A custom image processing algorithm was developed to track the concentrated sample in fluorescent images and then obtain the fluorescent signal intensity for quantitative analysis. As shown in Figure 4A,B, the original fluorescent image is first converted into a gray image.

The maximal gray value within the entire image is represented by max_*fluo*. In principle, the result of the sample concentration is relative to the gray value of the region with the concentrated sample which is normally larger than that of the background. A threshold value, *thres*, is defined to differentiate the region with the concentrated sample from the background,

(1) $$thres=\max\_fluo-\frac{\max\_fluo}{a}$$

where a∈[1.65, 1.95] is an empirical coefficient. Then, the gray value of each pixel in the gray image is redefined as follows, which further converts the original image into a binary image,

(2) $$fluo\_value(x,y)=\left\{ \begin{array}{ll} 255, & fluo\_value\left(x,y\right)\geq thres\\ 0, & fluo\_value\left(x,y\right)<thres \end{array}\right.$$

The converted binary image is shown in Figure 4C. The boundary for the region with the concentrated sample is determined by analyzing the binary image with Canny operator [31]. As shown in Figure 4D, the boundary of the region with the concentrated sample is marked with a red circle in the fluorescence image. The average gray value of all pixels within the determined region is calculated to represent the fluorescent signal intensity of the concentrated sample.

## 3. Results and Discussion

### 3.1. ICP Devices with Different Fabrication Methods

**Figure 5:**
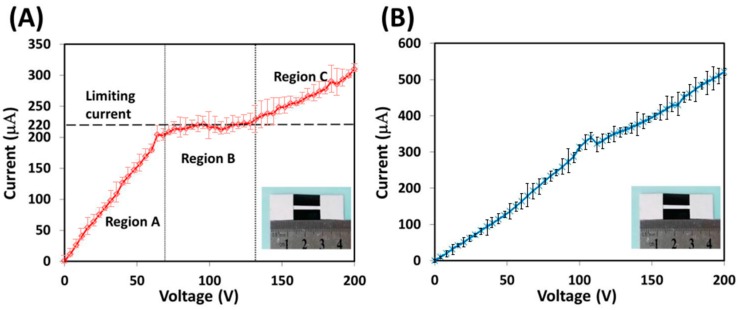
Current-voltage curves with two different types of ICP devices. (**A**) Type I; (**B**) Type II. The inset in A or B features a real ICP device.

As previously described, two types of paper-based ICP devices (type I and II), respectively, with two different methods for Nafion coating, were fabricated. For each ICP device, the middle channel made from Nafion-coated nitrocellulose membrane was cut into the dimensions of 2 mm × 16 mm. The two buffer pools were made from paper-based absorbent pads with the dimensions of 15 mm × 15 mm. The bottom layer was made from a black backing sheet with the dimensions of 20 mm × 50 mm. The width of the Nafion-coated region was around 2 mm, and the length was 2 mm. The distance from the Nafion-coated region to the closer buffer pool was around 2 mm. The real device is shown as an inset in Figure 5A or Figure 5B.

To discover the ICP efficiency of the developed device, before experimenting with the fluorescent dye sample, its electrokinetic characteristics were evaluated. First, each 300 µL of 10^−2^ M tris buffer was added to one of the two absorbent pads to establish two buffer pools. Then, a DC voltage was applied to the ICP device through two platinum electrodes which respectively contacted with two prewetted absorbent pads. The current-voltage (I-V) response for the two types of ICP devices was measured as the applied voltage was increased from 0 to 200 V in 50 discrete steps.

As shown in Figure 5, there is a significant difference between the two I-V response curves, respectively, with ICP devices type I and II. For the type I ICP device, the I-V curve (Figure 5A) consists of three distinct regions (A, B and C). Region “A” is known as the ohmic region where the current and voltage exhibit a linear relationship, which means that the conductance of the device remains constant and there is no ICP effect. Region “B” is known as the limiting region where the current remains almost constant around a limiting value when the voltage increases, which means that an ion-depletion effect occurs and a significant increase in the device resistance is caused because of the lack of ionic charges. Region “C” is known as the overlimiting region where the current increases nonlinearly with an increasing voltage, which means that the depletion zone starts to expand and the previously balanced ICP effect is broken. Similar to experimental results from other literature [18,20], the I-V response curve in Figure 5A confirms that ICP is successfully induced in the developed type I device. However, as shown in Figure 5B, for the type II ICP device, there are no three clear regions with the I-V response curve as exhibited in the type I ICP device. The current always increases with an increasing voltage within the entire curve, except for a short maintaining stage, which indicates that there is an insignificant or almost no ICP effect with the type II device. The above experiment was repeated at least three times, and similar results were obtained.

**Figure 6:**
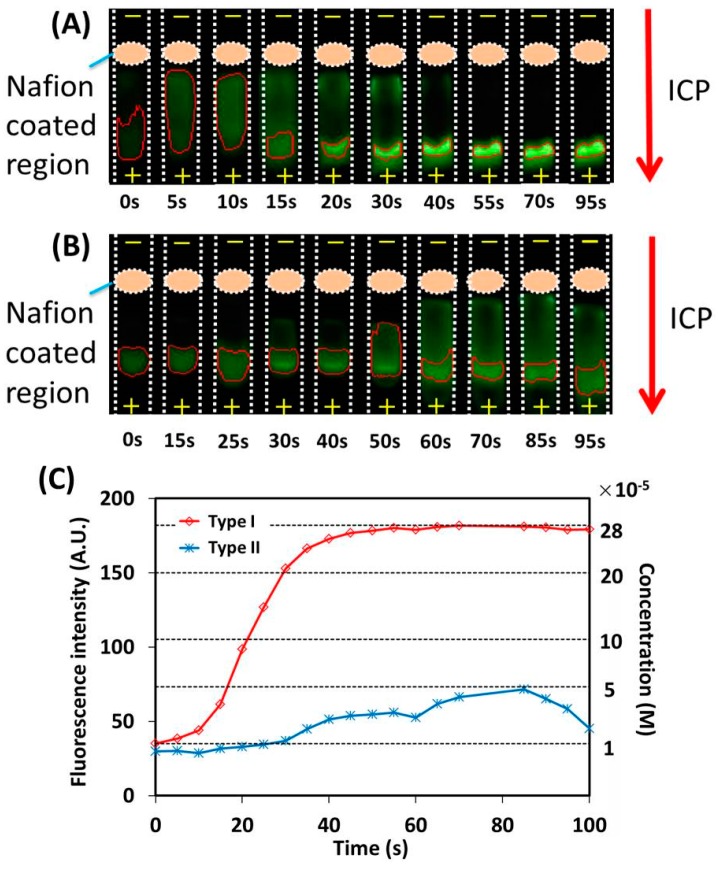
Experimental result with two types of ICP devices. (**A**) Fluorescence images captured at different times with ICP device type I; (**B**) Fluorescence images captured at different times with ICP device type II; (**C**) Variation of fluorescent signal intensity and corresponding sample concentration over time with two types of ICP devices.

In the next step, 10 μM of fluorescein isothiocyanate (FITC) was used as the fluorescent dye sample to evaluate the efficiency of the sample concentration with both types of ICP devices. To successfully induce the ICP effect with the developed device, 140 V was chosen as the driven voltage based on the above I-V curve. The process of sample concentration with the ICP effect was monitored in real time by a smartphone camera. Figure 6A,B present parts of the fluorescent images of the concentrated sample at different times from 0 to 100 s, respectively, for ICP devices type I and II. The region with the concentrated sample, which is marked with a red enclosed curve, is isolated and tracked by the developed custom image processing algorithm. The “+” and “−” signs in Figure 6A,B represent, respectively, anode and cathode electrodes. Figure 6C presents the variation of both the fluorescent signal intensity of the concentrated sample and the corresponding concentration over different times for both types of devices. The fluorescent signal intensity of the concentrated sample was obtained through quantitative analysis on the fluorescent images. A quadratic polynomial function was used to figure out the concentration of the concentrated sample from its fluorescent signal intensity.

As shown in Figure 6, for ICP device type I, the fluorescent signal intensity of the concentrated sample keeps increasing once the ICP effect has been induced, and finally a 28-fold maximal improvement in the sample concentration is obtained at 70 s. On the other hand, for ICP device type II, an insignificant sample concentration is observed and only a five-fold maximal improvement in the sample concentration is obtained. In terms of the increased fold in the sample concentration, it can be found that the efficiency of ICP device type I is significantly higher than that of type II. From the uniformity and porosity of the ion-selective nanostructures of the Nafion-coated region (scanning electron microscopy images in Figure 2), it can be inferred that improper heating to the Nafion-coated nitrocellulose membrane (ICP device type II) will most probably deteriorate the Nafion coating and hence decrease its ICP performance. The above experiment was repeated at least three times, and similar results were obtained. In principle, even a higher-fold maximal improvement in sample concentration can be obtained when the dimensions and sizes of the ICP devices are modified.

### 3.2. ICP Device with Adjusted Dimensions

**Figure 7:**
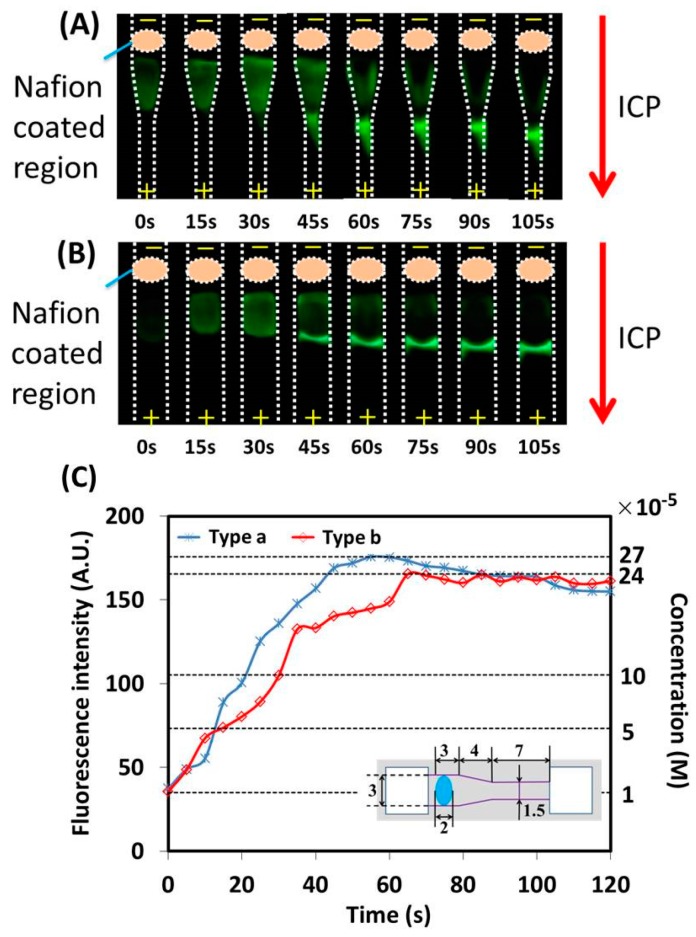
Experimental results with two types of ICP devices. (**A**) Fluorescence images captured at different times with ICP device type a; (**B**) Fluorescence images captured at different times with ICP device type b; (**C**) Variation of fluorescent signal intensity and corresponding sample concentration over time with two types of ICP devices. The insert in C features a schematic of ICP device type a.

To further improve the efficiency of the sample concentration with the ICP effect, an effort has been made by adjusting the dimensions of the ICP device. Specifically, a paper-based ICP device (named type a), which consists of two buffer pools and a non-uniform sample channel including wide, transitional and narrow parts, was fabricated to evaluate the performance of the device with adjusted dimensions. In detail, for each ICP device, the sample channel consists of one wide part (width: 3 mm, length: 5 mm), one transitional part (width: 3 to 1.5 mm, length: 4 mm) and one narrow part (width: 1.5 mm, length: 7 mm). The rest of the dimensions of the ICP device are similar to those of the previously described device. The schematic of the ICP device is shown as an inset in Figure 7C. To induce an efficient ICP effect, the Nafion coating method without heating was adopted here. For comparison, a normal ICP device (named type b) with the dimensions of 3 mm× 16 mm was fabricated, the width of which was larger than that of the ICP device (type I) fabricated in Section 3.1. Further, 10 μM of fluorescein isothiocyanate (FITC) was used as the fluorescent dye sample and 140 V was applied to the ICP device to provide a DC electrical field. Figure 7A,B present parts of the fluorescent images of the concentrated sample at different times from 0 to 120 s, respectively, for the evaluated ICP device (type a) and the normal one (type b). The “+” and “−” signs in Figure 7A,B represent, respectively, anode and cathode electrodes. The region with the concentrated sample is isolated and tracked by the developed custom image processing algorithm for quantitative analysis. Figure 7C presents the variation of both the fluorescent signal intensity of the concentrated sample and the corresponding concentration over different times.

As shown in Figure 7, for the ICP device (type a) with the adjusted dimensions, the fluorescent signal intensity of the concentrated sample increases once the ICP effect has been established, and finally a 27-fold maximal improvement in the sample concentration is obtained at 60 s. On the other hand, for the normal ICP device (type b), a 24-fold maximal improvement in the sample concentration is obtained at 65 s. For ICP device type a, the maximal fold of improvement in the sample concentration occurs when almost all of the sample reaches the narrow end of the transitional area of the non-uniform channel, and after that the increased fold slightly decreases. In terms of the increased fold in the sample concentration with the required time, the ICP device with adjusted dimensions shows higher efficiency than the normal device. The main reason for the improved efficiency is that the sample is physically concentrated when it moves from the wide channel to the narrow one. Because of different dimensions, the increased folds in the sample concentration with the normal ICP device, respectively, in Section 3.1 and Section 3.2 are different from each other.

### 3.3. ICP Device with Two Jointed Nitrocellulose Membrane Parts

**Figure 8:**
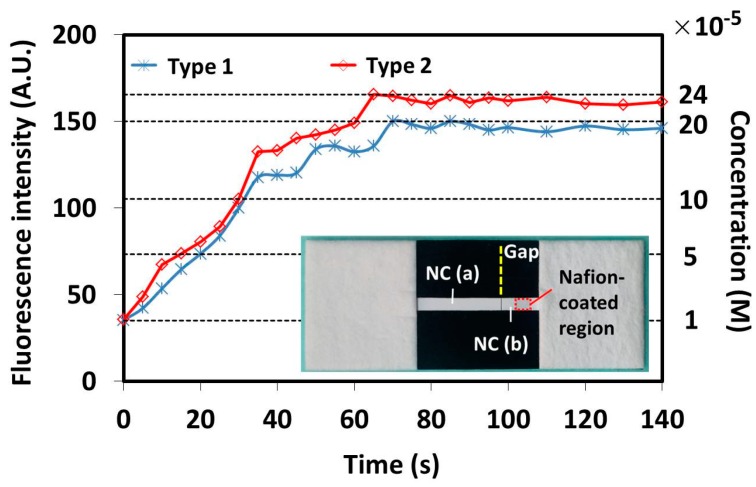
Variation of fluorescent signal intensity and corresponding sample concentration over time with two types of ICP devices. The inset features a real ICP device type 1.

It is desired to extend the application area of the ICP device by incorporating the ICP effect into the existing analytical devices to obtain fast and efficient separation and concentration of different biomolecules. Therefore, to explore the possibility of combining the ICP device with a traditional lateral flow strip or other µPADs, an ICP device (named type 1) whose channel was made from two jointed nitrocellulose membrane parts was fabricated and evaluated. Basically, the dimensions of the developed ICP device are similar to those of the previously described device except that there is a small gap (~0.2 mm) between the two jointed nitrocellulose membrane parts. The dimensions of the sample channel are 3 × 16 mm. The real device is shown as an inset in Figure 8. For comparison, a normal ICP device (named type 2) was fabricated. Figure 8 presents the variation of both the fluorescent signal intensity of the concentrated sample and the corresponding concentration over different times.

As shown in Figure 8, for the ICP device (type 1) with two jointed nitrocellulose membrane parts, finally a 20-fold maximal improvement in the sample concentration is obtained at 70 s. It was found that there was a solution leakage around the gap between the two parts of the paper strips. The solution leakage can work as a bridge to establish the integral solution layer for the ICP effect. Alternatively, for the normal ICP device (type 2), a 24-fold maximal improvement in the sample concentration is obtained at 65 s. In terms of the increased fold in the sample concentration with the required time, the efficiency of the ICP device with two jointed nitrocellulose membrane parts shows slightly lower efficiency than the normal one. However, although it is not as good as the normal one, the performance with the jointed ICP device is within the acceptable range. Therefore, it is demonstrated that the ICP effect will not be significantly deteriorated when the sample channel is made from two separated nitrocellulose membranes parts with a small gap in between. In the future, efforts can be made to develop an integrated analytical device based on the ICP effect.

## 4. Conclusions

This paper reports a microfluidic paper-based ICP device for sample concentration with smartphone detection. The microfluidic paper-based ICP device can be easily fabricated with a simple lamination method. To obtain the ICP effect, the nitrocellulose membrane, which is treated with a Nafion perfluorinated resin solution at one end, is used as the sample channel. Two buffer pools, which are made from paper-based absorbent pads, are used to prewet the sample channel through the capillary force to ensure that a proper electrical field will be established when a DC voltage is applied to the ICP device.

Smartphone detection is adopted for the real-time monitoring of the sample concentration with the ICP effect, which is beneficial to the potential application in point-of-care diagnosis. The fluorescent images of the concentrated sample are continuously collected at different times by a smartphone camera, and then processed by a custom algorithm for quantitative analysis. Beside the adjustment to the working current of LEDs, the smartphone also provides a friendly operational interface through custom application software developed with Java.

To successfully induce ICP, two different Nafion coating methods reported by the literature are evaluated and compared. With the experimental results, it has been demonstrated that improper heating to the Nafion-coated nitrocellulose membrane will deteriorate the Nafion coating and hence significantly decrease the ICP efficiency. To further improve the efficiency of the sample concentration due to the ICP effect, one type of ICP device with adjusted dimensions is fabricated and evaluated. It has been demonstrated that the efficiency of the sample concentration can be improved when the sample channel consists of multiple parts with different widths. To justify the feasibility of the integration of the ICP effect into the existing µPADs or traditional lateral flow strips, a combined ICP device consisting of two jointed nitrocellulose membrane parts with a small gap in between is fabricated and evaluated. Compared to the normal ICP device, a slightly lower efficiency of the sample concentration is obtained when the sample channel is made from two joint nitrocellulose membrane parts. In summary, a concise, simple and easy-to-operate ICP device with smartphone detection is developed for sample concentration, which is beneficial to improve the sensitivity of the paper-based analytical device at point-of-care diagnosis. With a small-size circuit for voltage amplification or a couple of batteries in a series to replace the bulky power supply, a portable microfluidic ICP-based system with smartphone detection can be developed for sample separation, concentration, or even diagnosis in settings with poor resources.
